# Medical Items

**Published:** 1912-09

**Authors:** 


### MEDICAL ITEMS

### DO YOU BELIEVE THE WORD OF SEVERAL THOUSAND PHYSICIANS?

If several thousand doctors told you that in PASADYNE, a distinctive tincture of passiflora Incarnata, they had found a most efficient substitute for chloral and the bromides, and that they had given up these latter drugs, would you believe them? While several thousand doctors will never tell you this, yet they could if an opportunity ever presented itself, for it is a fact. Gradually, during the last thirty-eight years, physicians, who have investigated the merits of PASADYNE (Daniel's Concentrated

Tincture of Passiflora Incarnata), have become users of it in preference to chloral and the bromides, for they have found it to possess just as much therapeutic activity as the drugs named and to be free from their dangerous after-effects. The possibility of habit-formation does not attach to the use of PASADYNE, nor is it depressing. It is the ideal sedative and soporific. A sample bottle will be furnished if application be made to the Laboratory of John B. Daniel, Atlanta, Ga.

---

THE POWER TO RECUPERATE

resident in the tissues, may be markedly augmented by Cord. Ext. Ol. Morrhuae Compound (Hagee), and with many physicians it is a routine practice to employ it for this purpose.

The usefulness of Cord Ext. Ol. Morrhuae Comp. (Hagee) as a reconstructive lies in the nutritious elements contained, which, when fed to impaired tissues build up and strengthen them. Each fluid ounce of the Cordial represents the extract obtainable from one-third fluid ounce of cod liver oil (the fatty portion being eliminated), 6 grains calcium hypophosphite, 3 grains sodium hypophospite with glycerine and aromatics. It is free from grease and the taste of fish.

---

The Pharmaceutical Department of this Company has this day taken over the Importation and Sale of the Modern Medicinal Preparations of the Farbwerke, vormals Meister Lucius & Bruening, Hoechst-on-Main, Germany, for which Victor Koechl & Co., and their immediate predecessors have been the American Agents for the past forty years.

The purpose of the change is to identify the name of Farbwerke, vormals Meister Lucius & Bruening more closely with their products.

The management of the Pharmaceutical Department of the Farbwerke-Hoechst Company will be the same as that of Victor Koechl & Co., so that the change is practically one in name only

*Ed. Journal-Record of Medicine, Atlanta, Ga.:*

We are pleased to announce that we have received stock of Neosalvarsan, a derivative of Salvarsan discovered by His Excellency Professor Paul Ehrlich.

Neosalvarsan possesses the following advantages over Salvarsan:

It dissolves readily in distilled water and forms a solution with a completely neutral reaction.

It is better tolerated and can be employed in larger doses than Salvarsan, while its potency is at least as powerful as that of Salvarsan.

Neosalvarsan is marketed according to doses which are equivalent to the dosage of Salvarsan::

0.15 gram Neosalvarsan marketed as Dose No. 1, equals 0.1 gram of Salvarsan.

0.3 gram Neosalvarsan marketed as Dose No. 2, equals 0.2 gram of Salvarsan.

0.45 gram Neosalvarsan marketed as Dose No. 3, equals 0.3 gram of Salvarsan.

0.6 gram Neosalvarsan marketed as Dose No. 4, equals 0.4 gram of Salvarsan.

0.75 gram Neosalvarsan marketed as Dose No. 5, equals 0.5 gram of Salvarsan.

0.9 gram Neosalvarsan marketed as Dose No. 6, equals 0.6 gram of Salvarsan.

For the intravenous injection of Salvarsan the average single doses for men are 0.4 to 0.5 gram and for women 0.3 to 0.4 gram; while the average single doses of Neosalvarsan for men are 0.6 to 0.75 to 0.9 gram and for women 0.45 to 0.6 to 0.75 gram. The maximum single dose for men being 1.5 gram and for women 1.2 gram, but treatment should not be commenced at the outset with such large doses. Solutions of Neosalvarsan must be injected immediately after their preparation, as they oxidize even more readily than the solutions of Salvarsan.

Very truly yours,

VICTOR KOECHL & CO.

New York, N. Y.

### HIGH-POTENCY ANTITOXIN.

A noticeable prefernce for concentrated anti-diphtheric serum (globulin), as compared with the older or "regular" form of diphtheria antitoxin, has manifested itself among the medical fraternity. "High potency, small bulk," appears to be the order of the day. A good index to the tendency in this direction may be found in the offerings of the manufacturers, who, as a matter of course, are promptly responsive to each new demand of the profession. For confirmation of the belief that the concentrated product is now in the ascendency, one has but to turn to the announcement of Parke, Davis & Co. in the current number of this journal, "Antotoxin That Justifies Your Confidence." Here one finds prominently featured the concentrated antidiphtheric serum (or globulin). It is interesting to note in this connection that a wider range of dosage than formerly is now offered—from 500 to 10,000 antitoxic units—the larger doses, of course, being provided for severe, late or other exceptional cases. And herein, at least, is one undisputed point in favor of the concentrated antitoxin: when a large dose is needed, it can be administered in this form without difficulty and with little danger of disturbance, owing to the comparative smallness of its bulk.

Some physicians, it may be noted, are under a misapprehension as to the nature of the concentrated antidiphtheric serum (globulin), assuming that it is widely different from this product which they have known for years as antidiphtheric serum. The idea is wholly erroneous. Concentrated antidiphtheric serum (globulin) is the regular product, precipitated and purified, from which most of the serum constituents have been eliminated except those bearing the antitoxin. It is in no sense inferior to the original serum—on the contrary, as previously noted, it possesses the advantage of lesser bulk.

### THE CONTROL OF PAIN.

The work of the conscientious physician is many sided and diverse, but no part or detail of his manifold duties is ever more obligatory or imperative than the control of pain. In the presence of physical suffering any other consideration than its prompt and positive relief, with rare exception, becomes of secondary importance. But insistent and pronounced as are the physician's duty always is to control and assuage the pains to which human flesh is subject, it should ever be his aim to accomplish this noble purpose in the best, as well as in the quickest possible way. Otherwise, with regard only for a patient's comfort, it is extremely liable that the agencies of relief will be attended by consequences serious in the extreme and not infrequently mor harmful in effect than the original pain itself.

The foregoing has particular significance for the cautious physician, inasmuch as he has in Papine a pain-relieving measure that enables him to control pain promptly and effectively, with the least possible untoward action. Representing as it does all the anodyne properties of the most potent opiate, but with the usual objectionable features reduced to a minimum, Papine is undoubtedly the most efficient analgesic at the command of the profession. Compared with the useful opiate, Papine will be found much more free from those disagreeable effects ordinarily considered inseparable from preparations of opium, such as constipation, nausea, gastro-intestinal derangement, and tendencies toward habit formation. Thus it can be employed in a wide variety of conditions which confidence, not alone in its anodyne and sedative action, but equally in its avoidance of disagreeable or unpleasant by-effects. In brief, Papine is the ideal preparation of opium, presenting all the advantages of this well-nigh indispensable drug with its nauseating, constipating and habit-forming tendencies reduced to a minimum. Innumerable physicians use it to the exclusion of all other opiates since it enables them to secure all the well-known benefits of the most potent opium derivative—with gratifying freedom from the usual disadvantages.

